# Modeling Light Response of Electron Transport Rate and Its Allocation for Ribulose Biphosphate Carboxylation and Oxygenation

**DOI:** 10.3389/fpls.2020.581851

**Published:** 2020-09-15

**Authors:** Zi-Piao Ye, Hua-Jing Kang, Ting An, Hong-Lang Duan, Fu-Biao Wang, Xiao-Long Yang, Shuang-Xi Zhou

**Affiliations:** ^1^ Maths and Physics College, Jinggangshan University, Ji’an, China; ^2^ Department of Landscape and Water Conservancy Engineering, Wenzhou Vocational College of Science and Technology, Wenzhou, China; ^3^ Jiangxi Provincial Key Laboratory for Restoration of Degraded Ecosystems & Watershed Ecohydrology, Nanchang Institute of Technology, Nanchang, China; ^4^ The New Zealand Institute for Plant and Food Research Limited, Hawke’s Bay, New Zealand

**Keywords:** photosynthesis, light response curve, electron flow partitioning, maximum *J*, saturation light intensity, ribulose biphosphate carboxylation, ribulose biphosphate oxygenation, model

## Abstract

Accurately describing the light response curve of electron transport rate (*J*–*I* curve) and allocation of electron flow for ribulose biphosphate (RuBP) carboxylation (*J*_C_–*I* curve) and that for oxygenation (*J*_O_–*I* curve) is fundamental for modeling of light relations of electron flow at the whole-plant and ecosystem scales. The non-rectangular hyperbolic model (hereafter, NH model) has been widely used to characterize light response of net photosynthesis rate (*A*_n_; *A*_n_–*I* curve) and *J*–*I* curve. However, NH model has been reported to overestimate the maximum *A*_n_ (*A*_nmax_) and the maximum *J* (*J*_max_), largely due to its asymptotic function. Meanwhile, few efforts have been delivered for describing *J*_C_–*I* and *J*_O_–*I* curves. The long-standing challenge on describing *A*_n_–*I* and *J*–*I* curves have been resolved by a recently developed *A*_n_–*I* and *J*–*I* models (hereafter, Ye model), which adopt a nonasymptotic function. To test whether Ye model can resolve the challenge of NH model in reproducing *J*–*I*, *J*_C_–*I* and *J*_O_–*I* curves over light-limited, light-saturated, and photoinhibitory *I* levels, we compared the performances of Ye model and NH model against measurements on two C_3_ crops (*Triticum aestivum* L. and *Glycine max* L.) grown in field. The results showed that NH model significantly overestimated the *A*_nmax_ and *J*_max_ for both species, which can be accurately obtained by Ye model. Furthermore, NH model significantly overestimated the maximum electron flow for carboxylation (*J*_C-max_) but not the maximum electron flow for oxygenation (*J*_O-max_) for both species, disclosing the reason underlying the long-standing problem of NH model—overestimation of *J*_max_ and *A*_nmax_.

## Introduction

Light intensity (*I*) is one of the most important environmental drivers affecting electron flow and its allocation for carboxylation versus oxygenation of ribulose biphosphate (RuBP). At *I* levels before reaching saturation intensity, the non-rectangular hyperbolic model (hereafter, NH model) is a sub-model which is widely used to characterize the light-response curve of electron transport rate (*J–I* curve) and to estimate the maximum *J* (*J*_max_) in C_3_ photosynthesis model (e.g., Farquhar et al., 1980; Farquhar and Wong, 1984; von Caemmerer, 2000; Farquhar et al., 2001; Long and Bernacchi, 2003; von Caemmerer et al., 2009; Bernacchi et al., 2013; Bellasio et al., 2015; Busch and Sage, 2017; Walker et al., 2017; Cai et al., 2018) and in C_4_ photosynthesis model (Berry and Farquhar, 1978; von Caemmerer and Furbank, 1999; von Caemmerer, 2013). At light saturation, *J*_max_ is estimated by the C_3_ photosynthesis model (Farquhar et al., 1980; von Caemmerer, 2013; Farquhar and Busch, 2017). Accurate estimation of *J*_max_ is important for understanding photosynthesis of C_3_ and C_4_ species. *J*_max_ is a key quantity to represent plant photosynthetic status under different environmental conditions when the net photosynthesis rate (*A*_n_) is limited by the regeneration of RuBP, associated with the partitioning of electron flow through photosystem II (PSII) for RuBP carboxylation (*J*_C_) versus that for RuBP oxygenation (*J*_O_) (Farquhar et al., 1980; Long and Bernacchi, 2003).

By simulating light-response curves of photosynthesis (*A*_n_ *–I* curve), NH model has been widely used to obtain key photosynthetic characteristics (e.g., the maximum net photosynthetic rate, *A*_nmax_; light compensation point when *A*_n_ = 0, *I*_c_; dark respiration rate, *R*_d_) for various species under different environmental conditions (e.g., Ögren & Evans, 1993; Thornley, 1998; Ye, 2007; Aspinwall et al., 2011; dos Santos et al., 2013; Mayoral et al., 2015; Sun et al., 2015; Park et al., 2016; Quiroz et al., 2017; Yao et al., 2017; Xu et al., 2019; Yang et al., 2020; Ye et al., 2020). Significant difference between observed *A*_nmax_ values and that estimated by NH model for various species has been widely reported (e.g., Chen et al., 2011; dos Santos et al., 2013; Lobo et al., 2014; Ogawa, 2015; Sun et al., 2015; Quiroz et al., 2017; Poirier-Pocovi et al., 2018; Ye et al., 2020). This long-standing challenge has been resolved by an *A*_n_–*I* model, which adopts a nonasymptotic function and can accurately reproduce *A*_n_–*I* curve over light-limited, light-saturated and photoinhibitory *I* levels (Ye et al., 2013) (hereafter, Ye model).

Recently, Buckley and Diaz-Espejo (2015) proposed that NH model would overestimate *J*_max_ due to its asymptotic function. A robust model which can accurately reproduce the observed *J–I* curve, and obtain *J*_max_, is urgently needed (Buckley and Diaz-Espejo, 2015). Furthermore, the light response of *J* partitioning for RuBP carboxylation and oxygenation (*J*_C_ *–I* and *J*_O_ *–I* curves), and the key quantities to describe the curves (e.g., the maximum *J*_C_, *J*_C-max_, and the maximum *J*_O_, *J*_O-max_, as well as their corresponding saturation light intensities) are rarely studied. Meanwhile, for the first time, we compared the performances of the two models in reproducing *J*_C_–*I* and *J*_O_–*I* curves.

This study aimed to fill these important gaps using an observation-modeling intercomparison approach. We firstly measured leaf gas exchange and chlorophyll fluorescence over a wide range of *I* levels for two C_3_ species [winter wheat (*Triticum aestivum* L.) and soybean (*Glycine max* L.)]. We then incorporated Ye model to reproduce *A*_n_–*I*, *J–I*, *J*_C_ *–I*, and *J*_O_ *–I* curves and return key quantities defining the curves, and evaluated its performance against NH model and observations.

## Materials and Methods

### Plant Material and Measurements of Leaf Gas Exchange and Chlorophyll Fluorescence

The experiment was conducted in the Yucheng Comprehensive Experiment Station of the Chinese Academy of Science. The detailed descriptions about soil and meteorological conditions in this experiment station were referred to Ye et al. (2019; 2020). Winter wheat was planted on October 4^th^, 2011 and the measurements were conducted on April 23^th^, 2012. Soybean was sown in on May 6^th^, 2013, and the measurements were performed on 27^th^ July, 2013. Using the Li-6400-40 portable photosynthesis system (Li-Cor, Lincoln, NE, USA), measurements on leaf gas exchange and chlorophyll fluorescence were simultaneously performed on mature fully-expanded sun-exposed leaves in sunny days. *J* was calculated as *J* = *Φ*_PSII_ × *I* × 0.5 × 0.84, where *Φ*_PSII_ is the effective quantum yield of PSII (Genty et al., 1989; Krall and Edward, 1992).

For soybean, *A*_n_–*I* curves and *J*–*I* curves were generated from applying different light intensities in a descending order of 2000, 1800, 1600, 1400, 1200, 1000, 800, 600, 400, 200, 150, 100, 80, 50, and 0 μmol m^-2^ s^-1^. For winter wheat, the light intensity gradient started from 1800 μmol m^-2^ s^-1^ as the maximum, in alignment with environmental light availability from October to April. At each *I* step, CO_2_ assimilation was monitored until a steady state was reached before logging a reading. Ambient CO_2_ concentration in the cuvette (*C*_a_) was kept constant at 380 μmol mol^-1^. Leaf temperature in the cuvette was kept at about 30°C for winter wheat and 36°C for soybean, respectively. The observation-modeling intercomparison was conducted within each species.

### *A*_n_ *–I* and *J–I* Analytical Models

NH model describes *J–I* curve as follows (Farquhar and Wong, 1984; von Caemmerer, 2000; von Caemmerer, 2013):

(1) $$J=\frac{\alpha_{\text{e}}I+J_{\text{max}}-\sqrt{{\left(\alpha_{e}I+J_{\text{max}}\right)}^{2}-4\alpha_{\text{e}}\theta J_{\text{max}}I}}{2\theta }$$

where *α*_e_ is the initial slope of *J–I* curve, *θ* is the curve convexity, *I* is the light intensity, and *J*_max_ is the maximum electron transport rate.

NH model describes *A*_n_ *–I* curve as follows (Ögren and Evans, 1993; Thornley, 1998; von Caemmerer, 2000):

(2) $$A_{\text{n}}=\frac{\alpha I+A_{\text{nmax}}-\sqrt{{\left(\alpha I+A_{\text{nmax}}\right)}^{2}-4\alpha \theta A_{\text{nmax}}I}}{2\theta }-R_{\text{d}}$$

where *α* is the initial slope of *A*_n_ *–I* curve, *A*_nmax_ is the maximum net photosynthetic rate, and *R*_d_ is the dark respiration rate when *I* = 0 μmol m^-2^ s^-1^. NH model cannot return the corresponding saturation light intensities for *J*_max_ or *A*_nmax_ due to its asymptotic function.

The model developed by Ye et al. (2013, 2019; hereafter, Ye model) describes *J–I* curve as follows:

(3) $$J=\alpha_{\text{e}}\frac{1-\beta_{\text{e}}I}{1+\gamma_{\text{e}}I}I$$

where *α*_e_ is the initial slope of *J–I* curve, and *β*_e_ and *γ*_e_ are the photoinhibition coefficient and light-saturation coefficient of *J–I* curve, respectively.

The saturation irradiance corresponding to the *J*_max_ (*I*_e_ *_-_*_sat_) can be calculated as follows:

(4) $$I_{\text{e}-\text{sat}}=\frac{\sqrt{(\beta_{\text{e}}+\gamma_{\text{e}})/\beta_{\text{e}}}-1}{\gamma_{\text{e}}}$$

Using Ye model, *J*_max_ can be calculated as follows:

(5) $$J_{\text{max}}=\alpha_{\text{e}}{\left(\frac{\sqrt{\beta_{\text{e}}+\gamma_{\text{e}}}-\sqrt{\beta_{\text{e}}}}{\gamma_{\text{e}}}\right)}^{2}$$

Ye model describes *A*_n_ *–I* curve as follows (Ye, 2007; Ye et al., 2013):

(6) $$A_{\text{n}}=\alpha \frac{1-\beta I}{1+\gamma I}I-R_{\text{d}}$$

where *α* is the initial slope of *A*_n_–*I* curve, *β* and *γ* are the photoinhibition coefficient and light-saturation coefficient of *A*_n_–*I* curve, respectively.

The saturation irradiance corresponding to *A*_nmax_ (*I*_sat_) can be calculated as follows:

(7) $$I_{\text{sat}}=\frac{\sqrt{(\beta +\gamma )/\beta }-1}{\gamma }$$

Using Ye model, *A*_nmax_ can be calculated as follows:

(8) $$A_{\text{nmax}}=\alpha \left(\frac{\sqrt{\beta +\gamma }-\sqrt{\beta }}{\gamma }\right)-R_{\text{d}}$$

### *J*_C_ and *J*_O_ Estimation and *J*_C_ *–I* and *J*_O_ *–I* Analytical Models

Combining measurements of gas exchange and chlorophyll fluorescence was a reliable and easy-to-use technique widely used to determine *J*_O_ and *J*_C_ (e.g., Peterson, 1990; Comic and Briantais, 1991). In C_3_ plants, carbon assimilation and photorespiration are two closely linked processes catalyzed by the key photosynthetic enzyme—RuBP carboxylase/oxygenase. Photorespiration is considered as an alternative sink for light-induced photosynthetic electron, and as a process helping consume extra photosynthetic electrons under high irradiance or other stressors limiting CO_2_ availability at Rubisco (Stuhlfauth et al., 1990; Valentini et al., 1995; Long and Bernacchi, 2003). When the other alternative electron sinks are ignored or kept constant, the electron flow is mainly allocated for RuBP carboxylation and RuBP oxygenation (e.g. Farquhar et al., 1980; von Caemmerer, 2000; Farquhar et al., 2001; Long and Bernacchi, 2003; von Caemmerer et al., 2009; Bernacchi et al., 2013; von Caemmerer, 2013), and *J*_C_ and *J*_O_ can be respectively calculated as follows (Valentini et al., 1995):

(9) $$J_{\text{C}}=\frac{1}{3}\left[J+8\left(A_{\text{n}}+R_{\text{day}}\right)\right]$$

(10) $$J_{\text{O}}=\frac{2}{3}\left[J-4\left(A_{\text{n}}+R_{\text{day}}\right)\right]$$

where *R*_day_ is the day respiration rate, and following Fila et al. (2006), *R*_day_ = 0.5 *R*_d_. In this study, *J*_C_ and *J*_O_ values calculated from Eqs. 9 and 10 were viewed as experimental observations—to be compared with modelled values derived from NH model and Ye model, respectively.

Using the same *J–I* modeling framework by Ye model, the light response of *J*_C_ (*J*_C_ *–I*) can be described as follows:

(11) $$J_{\text{C}}=\alpha_{\text{C}}\frac{1-\beta_{\text{C}}I}{1+\gamma_{\text{C}}I}I$$

where *α*_C_ is the initial slope of *J*_C_ *–I* curve, and *β*_C_ and *γ*_C_ are two coefficient of *J*_C_ *–I* curve. The maximum *J*_C_ (*J*_C-max_) and the saturation irradiance corresponding to the *J*_C-max_ (*I*_C_ *_-_*_sat_) can be calculated as follows:

(12) $$J_{\text{C}-\text{max}}=\alpha_{\text{C}}{\left(\frac{\sqrt{\beta_{\text{C}}+\gamma_{\text{C}}}-\sqrt{\beta_{\text{C}}}}{\gamma_{\text{C}}}\right)}^{2}$$

(13) $$I_{\text{C}-\text{sat}}=\frac{\sqrt{(\beta_{\text{C}}+\gamma_{\text{C}})/\beta_{\text{C}}}-1}{\gamma_{\text{C}}}$$

Using the same *J–I* modeling framework by Ye model, the light response of *J*_O_ (*J*_O_ *–I*) can be described as follows:

(14) $$J_{\text{O}}=\alpha_{\text{O}}\frac{1-\beta_{\text{O}}I}{1+\gamma_{\text{O}}I}I$$

where *α*_O_ is the initial slope of *J*_O_ *–I* curve, and *β*_O_ and *γ*_O_ are two coefficient of *J*_O_ *–I* curve. The maximum *J*_O_ (*J*_O-max_) and the saturation irradiance corresponding to the *J*_O-max_ (*I*_O_ *_-_*_sat_) can be calculated as follows:

(15) $$J_{\text{O}-\text{max}}=\alpha_{\text{O}}{\left(\frac{\sqrt{\beta_{\text{O}}+\gamma_{\text{O}}}-\sqrt{\beta_{\text{O}}}}{\gamma_{\text{O}}}\right)}^{2}$$

(16) $$I_{\text{O}-\text{sat}}=\alpha_{\text{O}}\frac{\sqrt{\beta_{\text{O}}+\gamma_{\text{O}}/\beta_{\text{O}}}-1}{\gamma_{\text{O}}}$$

Meanwhile, NH model can describe the *J*_C_ *–I* and *J*_O_ *–I* curves as follows:

(17) $$J_{\text{C}}=\frac{\alpha_{\text{C}}I+J_{\text{C}-\text{max}}-\sqrt{{\left(\alpha_{\text{C}}I+J_{\text{C}-\text{max}}\right)}^{2}-4\alpha_{\text{C}}\theta J_{\text{C}-\text{max}}I}}{2\theta }$$

where *α*_C_ is the initial slope of *J*_C_ *–I* curve, *θ* is the curve convexity, and *J*_C-max_ is the maximum *J*_C_, and

(18) $$J_{O}=\frac{\alpha_{O}I+J_{O-max}-\sqrt{{\left(\alpha_{O}I+J_{O-max}\right)}^{2}-4\alpha_{O}\theta J_{O-max}I}}{2\theta }$$

where *α*_O_ is the initial slope of *J*_O_ *–I* curve, *θ* is the curve convexity, and *J*_O-max_ is the maximum *J*_O_. NH model—Eqs. 17 and 18—cannot return the corresponding saturation light intensities for *J*_C-max_ or *J*_O-max_ due to its asymptotic function.

### Statistical Analysis

Statistical tests were performed using the statistical package SPSS 18.5 statistical software (SPSS, Chicago, IL). One-Way ANOVA was used to examine differences between parameter values estimated by NH model, Ye model and observed values of each parameter (*A*_nmax_, *I*_sat_, *J*_max_, *I*_e_ *_-_*_sat_, *J*_C-max_, *I*_C_ *_-_*_sat_, *J*_O-max_, *I*_O_ *_-_*_sat_, etc.). Goodness of fit of the mathematical model to experimental observations was assessed using the coefficient of determination (*R* ^2^ = 1 – SSE/SST, where SSE is the error sum of squares, and SST is the total sum of squares).

## Results

### Light Response of *A*_n_ and *J*

**Figure 1:**
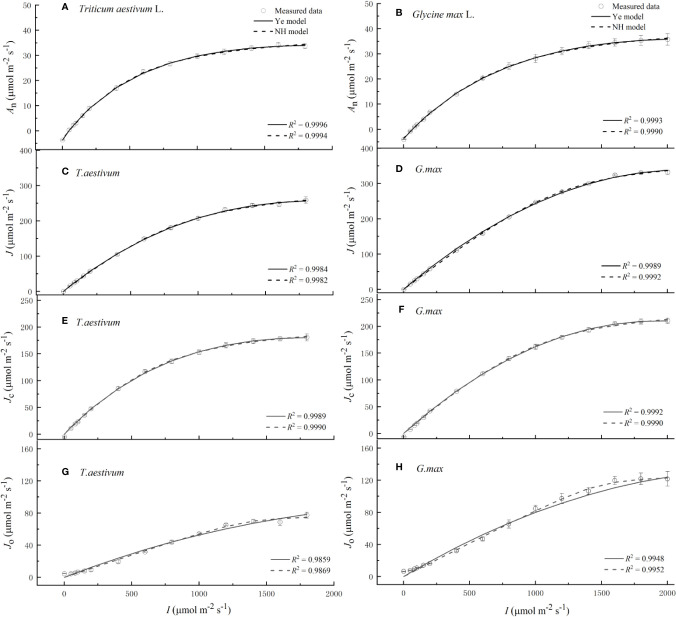
Light response curves of net photosynthetic rate **(A, B),** electron transport rate **(C, D),** electron flow for RuBP carboxylation **(E, F)** and the electron flow for RuBP oxygenation **(G, H)** for winter wheat (*Triticum aestivum* L.) and soybean (*Glycine max* L.), respectively, over the irradiance range from 0 to 2000 μmol m^−2^ s^−1^. Solid curves were fitted using Ye model, and dash curves were fitted using NH model. Values are means ± standard errors (*n* = 3).

Soybean and winter wheat exhibited an immediate and rapid initial increase of *A*_n_ (*α*) and *J* (*α*_e_) with the increasing *I* (**Figure 1** and **Table 1**). The increase of *A*_n_ and *J* continued until *I* reached the cultivar-specific maximum values (*A*_nmax_ and *J*_max_) at their corresponding saturation light intensities (*I*_sat_ and *I*_e_ *_-_*_sat_) (**Figure 1** and **Table 1**). Both NH model (Eqs. 1 and 2) and Ye model (Eqs. 3 and 6) showed high level of goodness of fit (*R* ^2^) to experimental observations of two species (**Figure 1** and **Table 1**). However, compared with observations, NH model significantly overestimated *A*_nmax_ and *J*_max_ (*P <* 0.05) for both soybean and winter wheat (**Table 1**). In contrast, *A*_nmax_ and *J*_max_ values returned by Ye model were in very close agreement with the observations for both species (**Table 1**).

**Table 1:** Fitted (Ye model and NH model) and measured values (Obs.) of parameters defining the light-response curve of photosynthesis (*A*_n_–*I* curve), electron transport rate (*J*–*I* curve), electron transport rate for RuBP carboxylation (*J*_C_–*I* curve), and electron transport rate for RuBP oxygenation (*J*_O_–*I* curve) for wheat and soybean species, respectively.

Parameters	*T. aestivum*			*G. max*		
	Ye model	NH model	Obs.	Ye model	NH model	Obs.
*A*_n_–*I* curve						
*θ* (dimensionless)	–	0.659 ± 0.046	–	–	0.644 ± 0.073	–
*α* (μmol μmol ^-1^)	0.077 ± 0.005^a^	0.069 ± 0.005^a^	–	0.059 ± 0.002^a^	0.055 ± 0.002^a^	–
*β* (m^2^ s μmol ^-1^)	(1.31 ± 0.07) × 10 ^-4^	–	–	(1.40 ± 0.08) × 10 ^-4^	–	–
*γ* (m^2^ s μmol ^-1^)	(1.02 ± 0.16) × 10 ^-3^	–	–	(5.76 ± 0.43) × 10 ^-4^	–	–
*A*_nmax_ (μmol m^-2^ s^-1^)	33.91 ± 1.14^b^	43.30 ± 1.28^a^	33.71 ± 1.12^b^	36.04 ± 2.11^b^	47.74 ± 2.08^a^	35.74 ± 2.29^b^
*I*_sat_ (μmol m^-2^ s^-1^)	1870.58 ± 26.45^a^	–	1799.59 ± 0.78^a^	2199.05 ± 78.46^a^	–	1999.73 ± 0.79^a^
*I*_c_ (μmol m^-2^ s^-1^)	50.08 ± 6.61^a^	50.42 ± 6.71 ^a^	50.20 ± 6.67^a^	66.72 ± 2.93^a^	67.38 ± 2.81^a^	66.82 ± 2.95^a^
*R*_d_ (μmol m^-2^ s^-1^)	3.60 ± 0.21^a^	3.29 ± 0.15^a^	3.73 ± 0.14^a^	3.76 ± 0.26^a^	3.58 ± 0.13^a^	4.03 ± 0.08^a^
Residuals	1.12 ± 0.15^a^	1.52 ± 0.34^a^	–	2.26 ± 0.14^a^	2.94 ± 0.84^a^	–
*J*–*I* curve						
*θ* (dimensionless)	–	0.816 ± 0.009	–	–	0.924 ± 0.005	–
*α*_e_ (μmol μmol ^-1^)	0.295 ± 0.012^a^	0.282 ± 0.012^a^	–	0.299 ± 0.006^a^	0.282 ± 0.005^a^	–
*β*_e_ (m^2^ s μmol ^-1^)	(2.42 ± 0.28) × 10 ^-3^	–	–	(3.07 ± 0.08) × 10 ^-4^	–	–
*γ*_e_ (m^2^ s μmol ^-1^)	(1.26 ± 0.66) × 10 ^-4^	–	–	(-1.50 ± 0.24) × 10 ^-4^	–	–
*J*_max_ (μmol m^−2^ s^−1^)	257.23 ± 7.36^b^	304.91 ± 7.11^a^	261.56 ± 7.32^b^	332.79 ± 5.16^b^	373.87 ± 5.47^a^	332.86 ± 5.01^b^
*I*_e-sat_ (μmol m^−2^ s^−1^)	1873.37 ± 109.46^a^	–	1734.16 ± 66.15^a^	1906.01 ± 19.97^a^	–	1933.23 ± 66.27^a^
Residuals	197.76 ± 119.18^a^	224.69 ± 81.52^a^	–	69.69 ± 6.00^a^	139.25 ± 19.30^a^	–
*J*_C_–*I* curve						
*θ* (dimensionless)	–	0.770 ± 0.040	–	–	0.871 ± 0.011	–
*α*_C_ (μmol μmol ^-1^)	0.266 ± 0.012 a	0.248 ± 0.014^a^	–	0.221 ± 0.003^a^	0.207 ± 0.002^b^	–
*β*_C_ (m^2^ s μmol ^-1^)	(2.07 ± 0.10) × 10 ^-4^	–	–	(2.54 ± 0.03) × 10 ^-4^	–	–
*γ*_C_ (m^2^ s μmol ^-1^)	(3.75 ± 0.75) × 10 ^-4^	–	–	(1.67 ± 1.37) × 10 ^-5^	–	–
*J*_C-max_ (μmol m^−2^ s^−1^)	180.49 ± 5.16^b^	210.90 ± 4.85^a^	182.48 ± 5.10^b^	210.66 ± 4.79^b^	242.42 ± 3.43^a^	210.76 ± 5.15^b^
*I*_C-sat_ (μmol m^−2^ s^−1^)	1813.42 ± 12.16^a^	–	1734.16 ± 66.15^a^	1938.65 ± 0.66^b^	–	1999.73 ± 0.79^a^
Residuals	72.25 ± 21.53^a^	62.74 ± 8.96^a^	–	78.54 ± 18.52^a^	83.50 ± 5.26^a^	–
*J*_O_–*I* curve						
*θ* (dimensionless)	–	0.839 ± 0.159	–	–	0.987 ± 0.008	–
*α*_O_ (μmol μmol ^-1^)	0.062 ± 0.007^a^	0.060 ± 0.007^a^	–	0.087 ± 0.005^a^	0.084 ± 0.005^a^	–
*β*_O_ (m^2^ s μmol ^-1^)	(3.45 ± 1.47) × 10 ^-4^	–	–	(4.12 ± 0.18) × 10 ^-4^	–	–
*γ*_O_ (m^2^ s μmol ^-1^)	(-1.98 ± 2.75) × 10 ^-4^	–	–	(-3.71 ± 0.31) × 10 ^-4^	–	–
*J*_O-max_ (μmol m^−2^ s^−1^)	85.67 ± 7.75^a^	91.67 ± 16.52^a^	79.08 ± 2.29^a^	124.34 ± 7.51^a^	127.13 ± 9.43^a^	121.61 ± 9.14^a^
*I*_O-sat_ (μmol m^−2^ s^−1^)	2790.82 ± 1085.62^a^	–	1734.16 ± 66.15^a^	1860.92 ± 34.19^a^	–	1866.73 ± 132.78^a^
Residuals	145.10 ± 57.72^a^	136.82 ± 60.25^a^	–	147.28 ± 14.61^a^	150.40 ± 13.62^a^	–

For A_n_–I curve, the parameters are: the initial slope of the A_n_–I curve (α_p_), the maximum A_n_ (A_nmax_) and the corresponding saturation irradiance (I_sat_), light compensation point (I_c_) and dark respiration rate (R_d_). For J–I curve, the parameters are: the initial slope of J–I curve (α_e_), the maximum J (J_max_) and the corresponding saturation irradiance corresponding to J_max_ (I_e-sat_). For J_C_–I curve, the parameters are: the initial slope of J_C_–I curve (α_C_), the maximum J_C_ (J_C-max_) and the corresponding saturation irradiance corresponding to J_C-max_ (I_C-sat_). For J_O_–I curve, the parameters are: the initial slope of J_O_–I curve (α_O_), the maximum J_O_ (J_O-max_) and the corresponding saturation irradiance corresponding to J_O-max_ (I_O-sat_). The observation-modeling intercomparison was only conducted within each species. Within each species the different the letters denote statistically significant differences between the values fitted by Ye model, NH model and measured values (Obs.) for each parameter (P ≤ 0.05). Values are the mean ± standard errors (n = 3).

### Light Response of *J*_C_ and *J*_O_

Both species exhibited an immediate and rapid initial increase of *J*_C_ (*α*_C_) with the increasing *I* (**Figure 1** and **Table 1**). The increase of *J*_C_ continued until *I* reached the cultivar-specific maximum values (*J*_C-max_) at the corresponding saturation light intensity (*I*_C_ *_-_*_sat_) (**Figure 1** and **Table 1**). Both Ye model (Eq. 11) and NH model (Eq. 17) showed high level of goodness of fit (*R* ^2^) to experimental observations of both species (**Figure 1** and **Table 1**). However, compared with observations, NH model significantly overestimated *J*_C-max_ (*P <* 0.05) for both soybean and winter wheat (**Table 1**). In contrast, *J*_C-max_ values returned by Ye model were in very close agreement with the observations for both species (**Table 1**).

Compared to the light-response rapidness of *J*_C_, *J*_O_ exhibited a much slower initial increase (*α*_O_) with the increasing *I* (**Figure 1** and **Table 1**). No species showed significant difference between the observed value of *J*_O-max_ and that estimated by Ye model (Eq. 14) or NH model (Eq. 18) (**Table 1**). Both models showed high level of goodness of fit (*R* ^2^) to experimental observations of both species (**Figure 1** and **Table 1**).

## Discussion

Assessed with an observation-modeling intercomparison approach, the results in this study highlight the robustness of Ye model in accurately reproducing *A*_n_ *–I*, *J–I*, *J*_C_ *–I*, and *J*_O_ *–I* curves and returning key quantities defining the curves, in particular: *A*_nmax_, *J*_max_, *J*_C-max_, and *J*_O-max_. On the contrary, the NH model significantly overestimates *A*_nmax_, *J*_max_, and *J*_C-max_ (**Table 1**). For the first time, our study discloses the previously widely reported overestimation of *J*_max_ (and *A*_nmax_) by the NH model is linked to its overestimation of *J*_C-max_ but not *J*_O-max_.

The overestimation of *A*_nmax_ by NH model found in this study is consistent with the previous reports (e.g., Calama et al., 2013; dos Santos et al., 2013; Lobo et al., 2014; Ježilová et al., 2015; Mayoral et al., 2015; Ogawa, 2015; Park et al., 2016; Quiroz et al., 2017; Poirier-Pocovi et al., 2018; Ye et al., 2020). The accurate returning of *A*_nmax_ by Ye model found in this study is consistent with previous studies using Ye model for various species under different environmental conditions (e.g., Wargent et al., 2011; Zu et al., 2011; Xu et al., 2012a; Xu et al., 2012b; Lobo et al., 2014; Xu et al., 2014; Song et al., 2015; Chen et al., 2016; Ye et al., 2019; Yang et al., 2020; Ye et al., 2020). The robustness of Ye model has also been validated for microalgae observations, including four freshwater and three marine microalgae species (Yang et al., 2020). The Ye model reproduced the *A*_n_ *–I* response well for all microalgae species, and produced *I*_sat_ closer to the measured values than those by three widely used models for microalgae (Yang et al., 2020). Meanwhile, the overestimation of *J*_max_ by NH model found in this study supports Buckley and Diaz-Espejo (2015) in highlighting the demerit of the asymptotic function (i.e. NH model).

One key novelty of the present study is its evaluation of both asymptotic and nonasymptotic functions in describing the light response of electron flow allocation for carboxylation and oxygenation respectively (i.e. *J*_C_–*I* and *J*_O_–*I* curves). To the best of our knowledge, this is the first study which has experimentally evidenced the robustness of a nonasymptotic function (Eqs. 3, 11, 14) in accurately (1) reproducing *J–I*, *J*_C_ *–I*, and *J*_O_ *–I* curves and (2) returning *J*_max_, *J*_C-max_, and *J*_O-max_ values, as well as their corresponding the saturation light intensities. These novel findings are of significance for our understanding of light responses of plant carbon assimilation and photorespiration—both are catalyzed by RuBP carboxylase/oxygenase.

The findings, and the approach of bridging experiment and modeling, in the present study remain to be tested for (1) species of different plant function types and/or climatic origin, which could exhibit different response patterns (Ye et al., 2020) and (2) plant response to interaction of multiple environmental factors (e.g., temperature, rainfall pattern, soil type) involving fluctuating light. The explicit and consistent modeling framework and parameter definitions on light responses (i.e. *A*_n_ *–I*, *J–I*, *J*_C_ *–I*, and *J*_O_ *–I*)—combined with the simplicity and robustness—allows for future transparent scaling-up of leaf-level findings to whole-plant and ecosystem scales.

## Conclusions

Ye model can accurately estimate *A*_nmax_, *J*_max_, and *J*_C-max_ which the NH model would overestimate. Adopting an explicit and transparent analytical framework and consistent definitions on *A*_n_ *–I*, *J–I*, *J*_C_ *–I*, and *J*_O_ *–I* curves, this study highlights the advantage of Ye model over NH model in terms of (1) its extremely well reproduction of *J–I*, *J*_C_ *–I*, and *J*_O_ *–I* trends over a wide *I* range from light-limited to light-inhibitory light intensities, (2) accurately returning the wealth of key quantities defining *J–I*, *J*_C_ *–I*, and *J*_O_ *–I* curves, particularly *J*_max_, *J*_C-max_, *J*_O-max_, and their corresponding the saturation light intensities (besides *A*_nmax_ and *I*_sat_ of *A*_n_ *–I* curve), and (3) being transparent in disclosing that the previously widely reported but poorly explained problem of NH model—overestimation of *J*_max_ (and the maximum plant carboxylation capacity)—is linked to its overestimation of *J*_C-max_ but not *J*_O-max_. Besides, NH model cannot obtain their saturation light intensities corresponding to *J*_max_, *A*_nmax_, *J*_C-max_, and *J*_O-max_ due to its asymptotic function. This study is of significance for both experimentalists and modelers working on better representation of photosynthetic processes under dynamic irradiance conditions.

## Data Availability Statement

The raw data supporting the conclusions of this article will be made available by the authors, without undue reservation.

## Author Contributions

All authors contributed to the conception of the work. H-JK mainly performed the experiment. Z-PY and S-XZ drafted the original manuscript. All authors critically reviewed and revised the manuscript with new data sets and contributed substantially to the completion of the present study. All authors contributed to the article and approved the submitted version.

## Funding 

This research was supported by the Natural Science Foundation of China (Grant No. 31960054 and 31560069) and the Key Science and Technology Innovation Team Project of Wenzhou City (Grant No. C20150008).

## Conflict of Interest

S-XZ was employed by the company The New Zealand Institute for Plant and Food Research Limited.

The remaining authors declare that the research was conducted in the absence of any commercial or financial relationships that could be construed as a potential conflict of interest.
